# Outcome of tracheostomy timing on critically ill adult patients undergoing mechanical ventilation: a retrospective observational study

**DOI:** 10.1186/cc9579

**Published:** 2011-03-11

**Authors:** A Dhrampal, D Pearson, N Berry

**Affiliations:** 1 Norfolk and Norwich University Hospital, Norwich, UK

## Introduction

Tracheostomy is now an established standard of care in the management of some critically ill patients. Despite this, however, the effect of its timing on patient outcome remains unclear [1].

## Methods

We interrogated the database of our clinical information system (MetaVision, iMDSoft) and identified 75 patients who underwent tracheostomy insertion. Outcome data, including 28-day mortality, length of stay (LOS) and weaning interval, were captured for those patients undergoing tracheostomy <4 days into critical care admission (early group) and >4 days into critical care admission (late group). Continuous data when expressed as mean (SD) were analysed using *t*-test and when expressed as median (IQR) were analysed using the Mann-Whitney U test. Binary outcome data were analysed using the chi-square test. *P *< 0.05 was considered statistically significant.

## Results

The early group (*n *= 32) had a mean LOS of 19 days (SD = 16.57), median weaning interval of 9 days (IQR = 9.5) and a mortality of 12.5% (*n *= 4). The late group (*n *= 43) had a mean LOS of 21.6 days (SD = 12.62), median weaning interval of 8 days (IQR = 13) and a mortality of 27.9% (*n *= 12). More tracheostomies were performed late at our institution, but despite this there was no significant difference in LOS (*P *= 0.481, *t *test), weaning interval (*P *= 0.852, Mann-Whitney U test) or 28-day mortality (*P *= 0.107, chi-square test) between the two groups.

## Conclusions

Many clinicians believe that early tracheostomy insertion may benefit critically ill patients requiring mechanical ventilation. This benefit does not seem to extent to 28-day survival, critical care LOS or weaning from mechanical ventilation.
